# Application of Vacuum-assisted Therapy in Postoperative Ascitic Fluid Leaks: An Integral Part of Multimodality Wound Management in Cirrhotic Patients

**Published:** 2007-04-16

**Authors:** S. Peter Stawicki, Naomi S. Schwarz, Sherwin P. Schrag, John J. Lukaszczyk, Mark E. Schadt, Anthony Dippolito

**Affiliations:** a Stawicki Research Foundation, King of Prussia, PA; b Temple University School of Medicine, Philadelphia, PA; c Department of Surgery, St. Lukes Hospital and Health Network, Bethlehem, PA

## Abstract

Surgery in patients with hepatic cirrhosis and ascites is associated with significant morbidity, including poor wound healing. Postoperative management of abdominal and perineal wounds in these patients poses a unique challenge owing to increased intra-abdominal pressure, risk for peritonitis, and ascitic fluid leakage. Vacuum-assisted closure (VAC) therapy reportedly improves angiogenesis and epithelialization, controls bacterial contamination, and removes excess tissue fluid. We present 4 cases of successful management of intractable postoperative ascitic fluid leaks utilizing VAC-based techniques. In one case, closure of a profusely draining perineal wound following an abdominoperineal resection was accomplished within 5 days of specialized VAC dressing application. In the other 3 cases, refractory drainage from midline laparotomy incision was successfully managed with the use of VAC therapy. In all 4 cases, the VAC-based system was effective in controlling drainage of ascites and subsequently sealing the wounds. Postoperative use of VAC in conjunction with optimization of medical therapy and judicious tapping of ascites provides a safe and effective method to control ascitic fluid leaks and promote definitive tissue sealing in patients with hepatic cirrhosis.

Vacuum-assisted closure (VAC, Kinetic Concepts, Inc, San Antonio, TX) system, based on subatmospheric pressure, has revolutionized the management of wounds.^1^,^2^ It has been successfully used in the setting of wounds complicated by infection, poor circulation, exposed bone or hardware, or previous dehiscence.^3^,^4^ VAC facilitates healing by reportedly improving the rate of angiogenesis, endothelial proliferation, the integrity of the capillary basement membranes, capillary blood flow, capillary caliber, and by decreasing interstitial edema and bacterial counts within the wound.^5^–^8^

Postoperative abdominal and perineal wounds in patients with hepatic cirrhosis and ascites represent a unique challenge. This is due to the presence of multiple complicating factors, including increased intra-abdominal pressure, ascitic fluid leaks, edema, and the increased risk of infection.^9^ In addition, ascites has been described as a risk factor not only for wound dehiscence but also for postoperative mortality.^10^,^11^

A method that would manage postoperative fluid leakage from the surgical wound, control bacterial contamination, and likely promote angiogenesis would be likely to promote wound healing and decrease postoperative complications in cirrhotic patients with ascites. Given the advantages of subatmospheric-pressure wound therapy, we examined its utility in the treatment of surgical patients with refractory ascitic leaks from their incisional wounds. We report 4 cases of this novel use of the VAC system.

## CASE 1

A 52-year-old man presented to the emergency department with acute upper-abdominal pain. His medical history included alcoholic hepatic cirrhosis complicated by severe ascites. Upon further evaluation, he was found to have free intraperitoneal air and was taken to the operating room. During exploration, an anterior gastric perforation was found in the antrum. Abdominal washout and Graham patch repair of the perforation were performed. The abdominal fascia was closed with a combination of running and interrupted sutures, followed by stapled skin closure.

The patient's hospital course was unremarkable until postoperative day 6, at which point he became febrile. His white blood cell count increased to 21,600 mm^−3^. Peritoneal fluid culture performed at that time demonstrated Staphylococcus species as well as *Escherichia coli*, and an antibiotic regimen of vancomycin, cefepime, and metronidazole was instituted. The patient's nutritional status was poor as evidenced by a prealbumin of 5.3 mg/dL, and concentrated nutritional supplements were started. Concurrently, the patient's abdominal girth continued to increase. On postoperative day 10, the midline incision started to leak ascitic fluid. This was initially addressed by instituting a regimen of diuretics and serial paracentesis procedures. Despite these interventions, the ascitic leak persisted.

**Figure 1:**
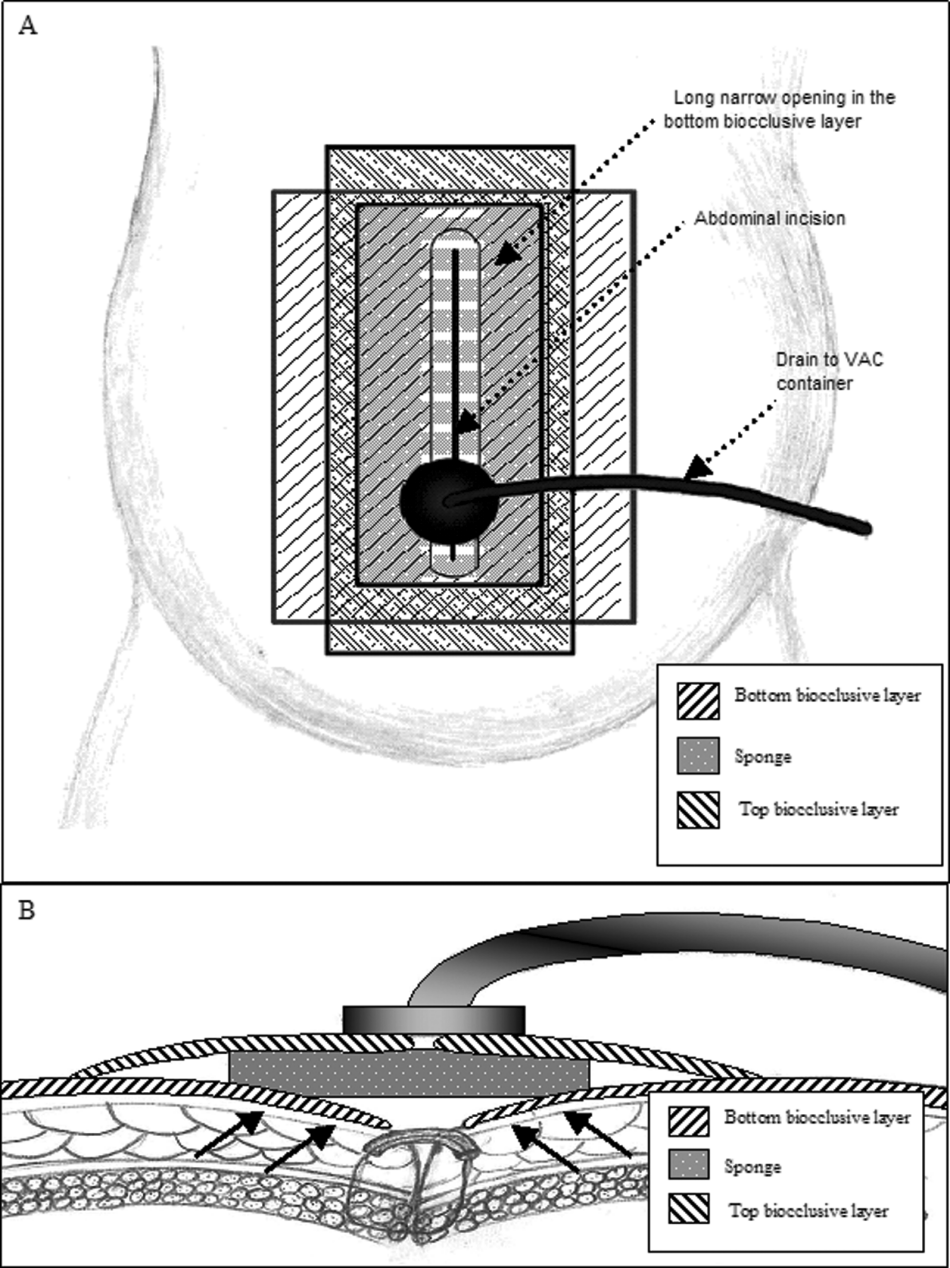
Depiction of VAC dressing used in Case 1. (A) Bottom layer of biocclusive was placed over patient's skin overlying the incision, and a slit was created over the incision site to allow for drainage. A sponge was placed over the biocclusive and incision. Finally, a top layer of biocclusive was placed over the sponge and adhering to the bottom biocclusive layer. A small cruciate incision was made in the top biocclusive, to which the VAC was attached. (B) Cross-section schematic view of the abdominal wall and VAC dressing. Proposed vectors of subatmospheric pressure forces are indicated by arrows.

A vacuum-assisted closure (VAC) dressing was applied on hospital day 14, hoping to control the leakage of ascitic fluid (Fig 1). Initially, the daily drainage was as high as 8 canisters, approximately 400 mL each. Despite this, the ascites were controlled with frequent canister and VAC dressing changes. The drainage decreased gradually to 1 canister per day prior to discharge on postoperative day 23.

In the meanwhile, the patient's peritonitis has resolved and his prealbumin level increased to 17.9 mg/dL, indicating improving nutritional status. He was discharged to home on a regimen of furosemide and spironolactone, and was provided with visiting nursing care to change the VAC dressing every 2 days or as needed. The VAC dressing was discontinued 7 days after discharge, with no further evidence of fluid leakage. He remains well at his 5-month clinic follow-up appointment.

## CASE 2

A 60-year-old man with alcoholic hepatic cirrhosis and ascites developed perianal epidermoid cancer, for which he underwent combined chemotherapy and radiation therapy with good initial response. Nine months later, he was found to have an enlarging lesion in his perianal region, which upon biopsy was shown to represent a recurrence. Abdominoperineal resection was performed, with malignancy-negative margins. At the time of this surgery, 4 L of ascites were evacuated. Because of prior radiation, the patient's perineal tissues were extremely frail. Although primary closure of the perineal wound was possible at the time of the abdominoperineal resection, the patient began to leak ascitic fluid through the wound soon on postoperative day 2.

On postoperative day 3, a reinforcing line of cutaneous sutures was placed in an attempt to control the process, but this ultimately proved inadequate. A concurrent medical therapy aimed at decreasing ascites was undertaken, and consisted of a regimen of diuretics and paracentesis sessions.

**Figure 2:**
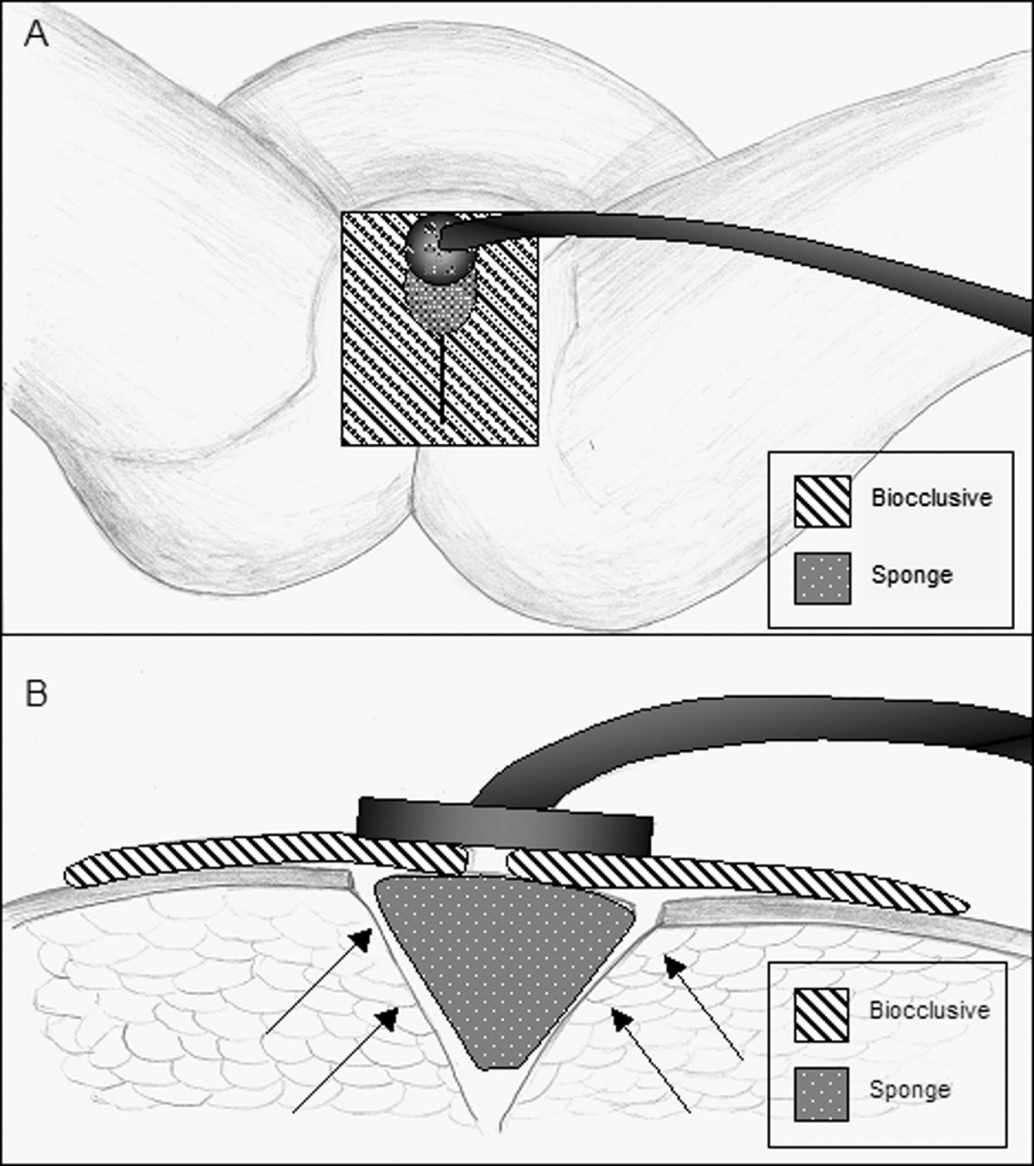
Depiction of VAC dressing used in Case 2. (A) Small sponge was placed over the anterior portion of the incision. This was covered with biocclusive, with a cruciate incision in the region overlaying the sponge. The VAC was attached with adhesive over this incision in the biocclusive. (B) Schematic cross section view of the VAC dressing. Proposed vectors of force are indicated by arrows.

By postoperative day 5, the leak was so severe that frequent bedside dressing changes (every 2–4 hours) were inadequate, and VAC dressing was applied to an open anterior area of the perineal wound (Fig 2). It was hoped that the VAC would contribute to closure by preferentially channeling the flow of ascitic fluid and protecting the remainder of the wound, and that the subatmospheric pressure would promote “collapse” of the perineal tissues and subsequent gradual wound closure.

Initially, it was hoped that the patient would be able to tolerate limited ambulation while on VAC therapy. However, every time he stood or sat up, more than 1 L of ascitic fluid drained with gravity and overwhelmed the drainage capacity of the VAC device and canister. With each such occurrence, complete VAC dressing change was required. Therefore, it was decided to place the patient on strict bedrest. This maneuver decreased the fluid drainage from 400 mL on day 2 to less than 30 mL on day 4 of VAC therapy. At this point, the patient was allowed to ambulate, and no further drainage was noted. The wound appeared to have sealed and was granulating well.

The patient was discharged to home on postoperative day 12, and continued to do well with continued VAC therapy as an outpatient. He had no further adverse events or any ascitic fluid leaks at the 3-month follow-up.

## CASE 3

A 61-year-old woman with liver failure secondary to autoimmune hepatitis was transferred to our facility owing to acute onset of epigastric pain, melanotic stools, and hypotension. Computed tomography of the abdomen and pelvis showed free intraperitoneal air as well as free fluid anterior to the stomach. Emergency laparotomy was performed and found both an anterior and a posterior antral ulcer. The patient underwent an antrectomy with Billroth II reconstruction. During the procedure, 3 L of bile-stained peritoneal fluid were evacuated. Three closed suction drains were left in place to drain the duodenal area, and the abdominal fascia was closed with a combination of running and interrupted sutures. The skin was closed with skin staples.

**Figure 3:**
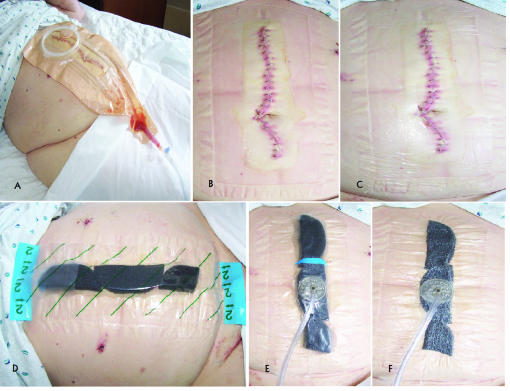
Stepwise depiction of VAC dressing placement in Case 3. (A) Wound manager overlying the midline wound prior to removal. (B) Midline wound following removal of wound manager and prior to VAC placement. (C) The bottom layer of biocclusive placed on the skin around the incision, with the incision itself left uncovered by biocclusive. (D) VAC sponge placed over the midline incision, along with the second biocclusive (indicated by green diagonal lines). (E) VAC suction device placed over biocclusive prior to application of subatmospheric pressure therapy. (F) The VAC dressing after institution of subatmospheric pressure therapy.

The patient initially did well, and 2 of the 3 drains, which were draining approximately 300 mL each day, were discontinued on postoperative days 3 and 5, respectively. However, the only remaining drain subsequently increased in output to more than 1 L daily. On postoperative day 7, large amounts of serous fluid started to leak from the midline incision site. The drainage measured more than 3 L on postoperative day 8. Over the next 4 days, the drain output decreased to less than 100 mL daily, and the drain was discontinued. Owing to continued leakage of ascites from the midline abdominal wound, a wound manager device was placed to control the leakage (Fig 3A).

Meanwhile, a regimen of spironolactone and furosemide was started in order to control the ascites medically, but the fluid drainage persisted. Concurrently, the patient was started on concentrated nutritional supplementation. On postoperative day 12, a VAC dressing was placed over the wound in an attempt to control the ascitic fluid leak (Figs 3B–3F). The VAC drained 1 L of serous fluid during the first 24 hours. However, there was minimal VAC drainage thereafter. Fifteen days following the operation, the patient was discharged to home. Outpatient nursing care was provided to help manage VAC dressing changes at home. The patient's medical management of ascites consisted of a continued regimen of spironolactone. She was doing well at the 2-month follow-up visit, with no evidence of any recurrent drainage of ascites.

## CASE 4

A 50-year-old woman presented to the emergency department with diffuse, moderately severe abdominal pain, worse in the left lower quadrant. She has a known history of alcoholic hepatic cirrhosis and moderate ascites. The patient was found to have free intraperitoneal air and was taken for an emergency laparotomy.

Intraoperatively, she was found to have a descending colonic perforation due to diverticular disease. Because of the patient's extremely poor general condition and ongoing abdominal sepsis and hemodynamic instability, the patient required an abbreviated colon resection, with a period of intensive care unit resuscitation before returning to the operating room for creation of an end-colostomy. Following the second operation, the patient slowly recovered from the severe sepsis and multiorgan failure and her activity level was being gradually increased. However, on postoperative day 7, she was noted to have increased abdominal girth, which coincided with increased drainage from her midline abdominal wound.

The quality of the fluid was consistent with ascites, and therapy with spironolactone and furosemide was initiated. The leakage persisted, however, necessitating placement of a wound management device over the entire wound. After 3 days of continued diuretic therapy, with an addition of paracentesis, wound drainage in excess of 1 L daily persisted.

In an attempt to control the drainage, a VAC dressing was applied over the entire length of the abdominal wound on postoperative day 10, resulting in nearly immediate decrease of the ascitic leak to approximately 150 mL/d. Over the next 7 days, with continued medical therapy and 2 sessions of paracentesis, the wound drainage decreased to less than 30 mL/d, and the VAC therapy was discontinued prior to patient discharge from hospital. There was no further drainage from the abdominal wound at the 6-week follow-up.

## DISCUSSION

Vacuum-assisted closure technology has a wide range of applications in wound management. The number of indications for VAC therapy is quickly growing.^3^,^4^ The beneficial effect of VAC therapy on tissue vasculature, epithelialization, edema, and infection has been shown to expedite healing of many wound types.^5^,^8^

Morbidity and mortality in patients with hepatic cirrhosis and ascites is due to numerous factors, including wound dehiscence, peritonitis, sepsis, liver failure, gastrointestinal bleeding, increased risk of pneumonia, and poor nutritional state.^12^ The 2 variables that can be controlled by the application of VAC therapy are (a) bacterial contamination of the wound and (b) enhanced wound vascularity due to reportedly beneficial effects of VAC therapy on tissue angiogenesis, capillary blood flow, and caliber. Combined, these factors may promote wound healing and decrease the risk of wound infection and/or dehiscence.^3^,^5^–^8^,^13^ In addition, the subatmospheric pressure exerted on the wound by VAC therapy may help elevate and approximate wound edges, resulting in quicker sealing of the wound and decreased leakage of ascitic fluid.

Patient must be carefully followed while on VAC therapy to avoid significant fluid loss and to closely monitor an already tenuous electrolyte balance in the setting of liver failure. The added benefit of VAC therapy is the ability to accurately quantify fluid drainage, which in turn allows more accurate estimation of fluid replacement requirements. For cases described in this report, the standard replacement fluid for both paracentesis and VAC-evacuated ascites was albumin given at between 5 and 8 g/L of ascites.^14^ Diagnostic and therapeutic paracentesis, diuretic therapy, and low-salt diets should be continued during the postoperative period in these patients.^15^

The 4 patients in this series who underwent VAC therapy applied for persistent ascitic fluid leaks from surgical wounds recovered very well. The application of VAC therapy in these patients resulted in decreased ascitic fluid leakage, sealing of the wound edges, and ultimately wound closure. It also appears that VAC therapy is more manageable by both patients and nurses than conventional wound therapy. Improved patient comfort and lower requirement for nursing care resulted from less frequent dressing changes and better control of the ascitic fluid flow (ie, prevention of noncontained leaks), allowing for earlier and more frequent patient mobilization. In fact, early mobilization was possible in 3 of the 4 cases, with the perineal wound patient (case number 2) requiring an initial 4-day period of bed rest. Furthermore, the speed of recovery and the ability to return to full activity due to the benefits of VAC therapy in this group of patients are impressive.

In light of the 4 cases presented in this report, we believe that utilization of appropriately fashioned VAC dressings in the setting of postoperative ascitic leaks may present physicians with a new option for management of patients with hepatic cirrhosis and ascites. This technology, in conjunction with optimization of diuretic and nutritional therapies, as well as judicious use of paracentesis, provides a safe and effective method of postoperative ascitic leak management in the setting of hepatic cirrhosis.

## CONCLUSION

Vacuum-assisted closure therapy appears to improve wound healing and closure in the setting of postoperative ascitic leaks and hepatic cirrhosis. It should be considered as part of a multimodality treatment including paracentesis, medical therapy, and aggressive nutritional support. Vacuum-based therapy appears to be safe, effective, and convenient to the patient and nursing staff, and allows for less frequent dressing changes and better quantification of fluid loss from the wound.
